# Hypovitaminosis D in healthy children in Central Thailand: prevalence and risk factors

**DOI:** 10.1186/s12889-015-1588-6

**Published:** 2015-03-14

**Authors:** Kanit Reesukumal, Kotchamol Manonukul, Orathai Jirapongsananuruk, Wijittra Krobtrakulchai, Sithikan Hanyongyuth, Somruedee Chatsiricharoenkul, Busadee Pratumvinit

**Affiliations:** Department of Clinical Pathology, Faculty of Medicine Siriraj Hospital, Mahidol University, 2 Wang Lang Road, Bangkok Noi, Bangkok 10700 Thailand; Department of Pediatrics, Faculty of Medicine Siriraj Hospital, Mahidol University, Bangkok, Thailand; Department of Pharmacology, Faculty of Medicine Siriraj Hospital, Mahidol University, Bangkok, Thailand

**Keywords:** 25-Hydroxyvitamin D, Hypovitaminosis D, Vitamin D insufficiency, Vitamin D deficiency, Children

## Abstract

**Background:**

There are limited data regarding the prevalence and risk factors relating to hypovitaminosis D in children of Thailand, a tropical country with abundant sunlight. The objective of this study was to assess the prevalence of hypovitaminosis D and examine factors associated with hypovitaminosis D in school-aged children in Bangkok, Thailand – a centrally located capital city.

**Methods:**

This cross-sectional study evaluated 159 healthy children (33.3% boys and 66.7% girls), aged 6 to 12 years, in Bangkok, Thailand (located at 13.45°N). Fasting plasma samples were examined for total 25-hydroxyvitamin D [25(OH)D] using electrochemiluminescence immunoassay. Demographic characteristics (age, sex, household income), past medical history (birth weight, allergic diseases, hospitalization), amount of sun exposure, anthropometric data, and selected biochemical tests were used to investigate for factors associated with hypovitaminosis D.

**Results:**

Overall, the mean ± SD level of plasma 25(OH)D was 64.0 ± 15.1 nmol/L. Hypovitaminosis D (< 75 nmol/L) was presented in 79.2% of subjects. Of these, the prevalence of vitamin D insufficiency and vitamin D deficiency were 59.7% and 19.5%, respectively. In univariate analysis, children with hypovitaminosis D (< 75 nmol/L) had a higher mean body mass index (BMI) percentile than the vitamin D-sufficient group (56.7 ± 33.9 vs. 42.6 ± 36.0; *P*-value = 0.04). Plasma PTH levels in the children with hypovitaminosis D were significantly higher than in the children with normal levels of vitamin D (4.34 ± 1.38 vs 3.78 ± 1.25 pmol/L; *P*-value = 0.04). In multivariate analysis, high BMI percentile and high PTH concentration were the parameters associated with 25(OH)D level < 75 nmol/L.

**Conclusion:**

The prevalence of hypovitaminosis D in healthy Thai children is very high, despite their exposure to sunlight, and that prevalence increases in children with a high BMI percentile. As a result, a formal recommendation for vitamin D supplementation in Thai children should be considered.

## Background

Vitamin D is important for calcium absorption from the gut and for promoting bone growth during childhood [1]. The most evident manifestation of vitamin D deficiency in infants and children is nutritional rickets [2]. Vitamin D receptors are present in most cells and tissues in the body, including osteoblasts, immune cells, β-islet cells, brain, heart, skin, gonads, prostate, colon, and breast [3]. Several studies have demonstrated the role of vitamin D in decreasing the risk of many chronic illnesses, including common cancers [4,5], autoimmune diseases [6,7], infectious diseases [8,9], and cardiovascular diseases [10].

Vitamin D is absorbed from dietary food and produced by the skin during exposure to sunlight. People living near the equator who are exposed to sunlight without sun protection should have robust levels of 25-hydroxyvitamin D [25(OH)D]. However, recent studies have shown that hypovitaminosis D in children is very high, worldwide [11,12]. Yet, limited data are available regarding the vitamin D status of children living in Bangkok (geographically located at 13.45°N), the capital of Thailand, where vitamin D supplements are not currently recommended and a vitamin D food fortification policy has not been implemented.

The aim of the present study was to evaluate the prevalence of hypovitaminosis D in Thai schoolchildren and investigate the factors associated with hypovitaminosis D. The results of this study may indicate the necessity of vitamin D supplementation for children in Thailand.

## Methods

### Subjects

This cross-sectional study was conducted amongst 1,268 children who attended a public primary school in Bangkok, Thailand. Bangkok is located at the latitude of 13°45′N, a location that is, in part, characterized by abundant sunlight all year round. However, there are variations in ultraviolet (UV) irradiances in different months of the year. In Nakhon Pathom, which is located near Bangkok in the central part of Thailand, UV irradiances are highest in March or April, stable from May to September, lower from October to December, and then have an increasing trend from January to April [13].

Prior to commencing, this study was approved by the Siriraj Institutional Review Board. Written informed consent was obtained from all parents and written assent was obtained from all children.

Healthy children aged 6 to 12 years were enrolled between December 2011 and February 2012. Exclusion criteria were: liver or kidney diseases, malabsorptive disorders, previous surgery of gastrointestinal tract, tuberculosis, and use of steroids or anticonvulsants within the past 6 months. All participants or guardians completed questionnaires for the purpose of providing socio-demographic data, medical history, and amount of sun exposure. The amount of sun exposure was evaluated by two questions. One question inquired about the body part(s) exposed to the sun from 10 a.m. to 3 p.m. [14] without sun protections, such as sunscreen, umbrella, and/or clothing. The second question asked about estimated weekly sun exposure time. Body surface area was calculated using Lund and Browder chart [15].

A previous study regarding prevalence of hypovitaminosis D in Kuala Lumpur, Malaysia (latitude 3.09°N), a country which is located near Thailand, revealed a prevalence of 72.4%, when using the cut-off point of ≤ 50 nmol/L [16]. Using an estimated prevalence of 72% in Thai school children with a 7% margin of error, a total sample size of 158 school children was calculated for this study.

### Anthropometric measurements

For anthropometric measurements, the participants wore light clothing and no shoes. Body weight and body composition (body fat, bone mass, and muscle mass) were measured during the same time period (between the hours of 7 a.m. and 10 a.m.) using Tanita BC-541 Inner Scan Body Composition Monitor (Tanita Corporation, Japan). Body weight was measured to the nearest 0.1 kg. Body composition was measured using bioelectrical impedance analysis and body fat percentile was acquired from reference curves by McCarthy et al. [17]. Height was recorded to the nearest 0.1 cm using the Leicester Height Measure (Child Growth Foundation (CGF), London, England, UK). Each participant’s body mass index percentile (BMI) was calculated using the Centers for Disease Control and Prevention BMI tool [18]. Waist and hip circumference were measured using a non-stretchable tape measure. Waist circumference was measured at the minimum circumference between the iliac crest and the rib cage. Hip circumference was measured at the maximum protuberance of the buttocks; the waist-hip ratio (WHR) was then calculated.

### Blood analysis

Overnight fasting blood samples were collected from all participants between the hours of 7 a.m. and 10 a.m. during the month of February 2012. A team consisting of medical technologists to perform phlebotomy, researchers, and a physician was present during each patient blood sampling and anthropometric measurement session. A total of 10 mL venous blood was collected into two tubes: 3 ml in an ethylenediaminetetraacetic acid (EDTA)-containing tube for the determination of intact parathyroid hormone (PTH) and total 25-hydroxyvitamin D (25(OH)D) concentrations and another 7 ml in a tube containing lithium heparin for the analysis of calcium, phosphorus, magnesium, and albumin. Plasma parathyroid hormone (PTH) and total 25(OH)D were analyzed using electrochemiluminescence immunoassay on Elecsys 2010 analyzers (Roche Diagnostics, Mannheim, Germany). Plasma calcium, phosphorus, magnesium, and albumin were analyzed using Modular P800 (Roche Diagnostics, Mannheim, Germany). The coefficients of variation (CVs) for 25(OH)D were 7.07% at a mean level of 40.3 nmol/L and 4.63% at a level of 84.5 nmol/L. The CVs were 2.28% to 2.30% for PTH and 0.7% to 2.3% for albumin and cations. Total 25(OH)D was reported in nanomoles per liter (nmol/L) and the conversion factor to conventional unit (ng/mL): 1 nmol/L = 0.40 ng/mL.

### Statistical analysis

All analyses were performed using PASW v. 18.0 software (SPSS Inc, Chicago, IL, USA) and Microsoft Excel 2007 (Microsoft Corp, Redmond, WA). A two-sided *P*-value of 0.05 was considered to be statistically significant. Continuous variables are expressed as mean ± SD or median (IQR). Categorical variables are expressed as proportions. The relationship between PTH and 25(OH)D concentration was assessed using Pearson’s correlation coefficient. We used the Mann–Whitney *U* test to compare the mean of the product of BSA with the duration of sunlight exposure per week (% BSA × h/week) values between BMI percentile groups (< 85^th^ vs. ≥ 85^th^ percentile).

The following definitions for vitamin D status were used: vitamin D sufficiency, 25(OH)D level ≥ 75 nmol/L; hypovitaminosis D, 25(OH)D level < 75 nmol/L; vitamin D insufficiency, 25(OH)D level 50–74.9 nmol/L; and vitamin D deficiency; 25(OH)D level < 50 nmol/L [19,20]. Multiple logistic regression analysis for factors associated with hypovitaminosis D (25(OH)D < 75 nmol/L) was performed using variables with a *P*-value < 0.2 without multicollinearity in univariate analyses.

## Results

**Figure 1:**
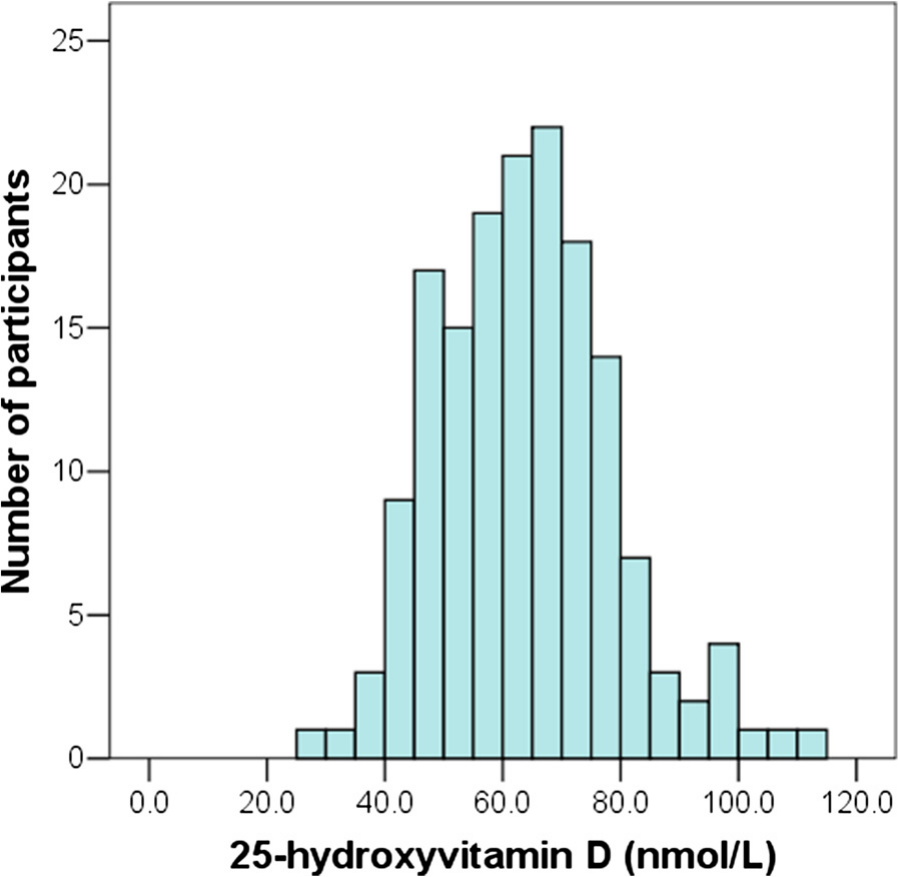
**Distribution of plasma 25-hydroxyvitamin D in healthy Thai school-aged children living in Central Thailand.**

One hundred and fifty-nine of 1,268 children with a mean age of 9.9 ± 1.6 years (age range: 6.5-12.8 years) participated in this cross-sectional study. There were 53 (33.3%) boys and 106 (66.7%) girls. The mean BMI percentile of participants was 53.7 ± 34.7. The mean 25(OH)D concentration for the entire group was 64.0 ± 15.1 nmol/L, ranging from 28.9 to 113.4 nmol/L (Figure 1).

**Figure 2:**
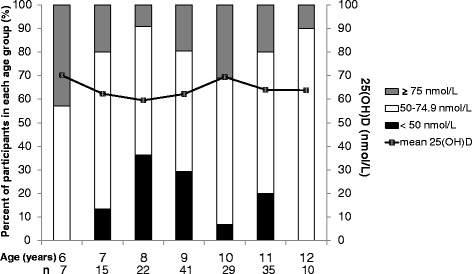
**Concentration of 25-hydroxyvitamin D by age.**

Subjects in the six-year-old age group (6–6.9 year) had the highest proportion of vitamin D sufficient status (42.8%) with a mean 25(OH)D concentration of 70.1 nmol/L, while children in the eight-year-old age group (8–8.9 year) had the lowest proportion (9.1%), with a mean 25(OH)D concentration of 59.5 nmol/L. No trend in vitamin D status was found among the different age groups (Figure 2). Overall, hypovitaminosis D was found in 126 (79.2%) subjects. Of these, 95 (59.7%) had vitamin D insufficiency and 31 (19.5%) had vitamin D deficiency. No subject had 25(OH)D level below 25 nmol/L. Thirty-nine of fifty-three boys (73.6%) had 25(OH)D levels lower than 75 nmol/L and 9 (17.0%) of those had 25(OH)D level lower than 50 nmol/L. Girls with 25(OH)D levels less than 75 and 50 nmol/L were 87 (82.1%) and 22 (20.8%), respectively. Vitamin D status was not significantly different between the two genders.

The mean PTH was 4.22 ± 1.37, ranging from 1.34 to 9.26 pmol/L. Four (2.52%) participants had elevated levels of PTH (> 6.89 pmol/L). Hyperparathyroidism was found in 3.2% (3/95) of subjects with vitamin D insufficiency and 3.2% (1/31) of subjects with vitamin D deficiency. Mean corrected total calcium was 2.37 ± 0.06 mmol/L, with one (0.63%) study participant having an elevated corrected calcium (> 2.5 mmol/L). The median (IQR) duration of sun exposure was 4.00 (1.5-11.3) hours per week. The median (IQR) value of the product of BSA and duration of sunlight exposure per week (% BSA × h/week) in overweight or obese subjects (BMI percentile ≥ 85^th^, n = 32) was not significantly different from that of healthy weight or underweight subjects (BMI percentile < 85^th^, n = 91) [133.8 (15.6-283.8) vs. 88.5 (29.5–309.8) m^2^h, *P* = 0.78].

**Figure 3:**
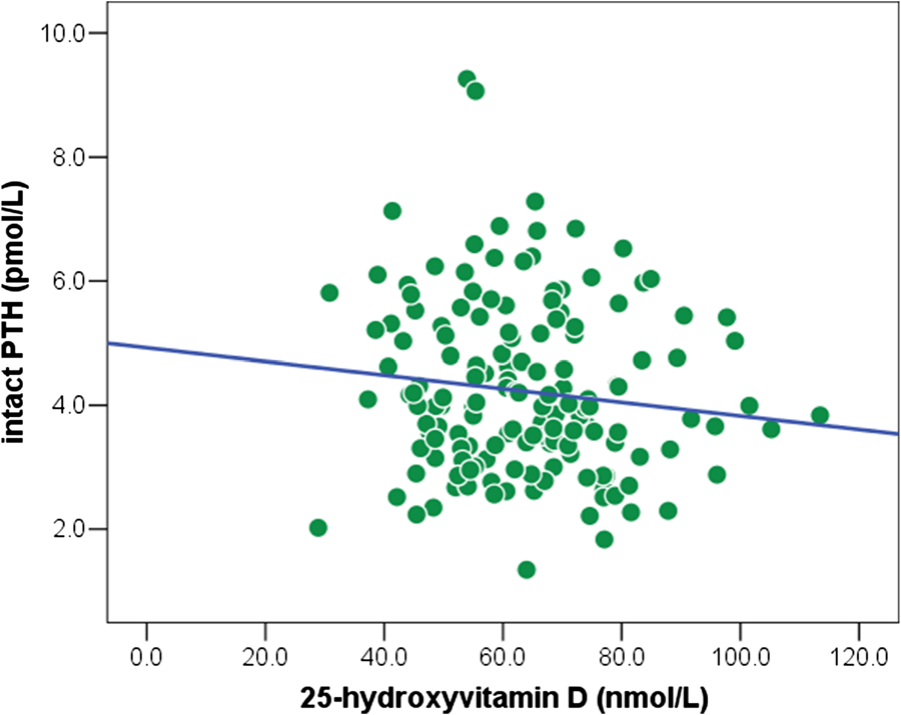
**Relationship between plasma levels of intact PTH and 25-hydroxyvitamin D in school children (n = 159); Pearson** ***r*** **= −0.12,** ***P-value*** **= 0.13.**

In univariate analysis, hypovitaminosis D children had a higher mean BMI percentile than vitamin D-sufficient children (56.7 ± 33.9 vs. 42.6 ± 36.0; *P*-value = 0.04) (Table 1). Waist circumference, body fat percentile, and muscle mass were higher in the hypovitaminosis D group; however, no statistically significant difference was observed (*P*-value = 0.07, 0.10, and 0.18, respectively). Body fat percentage was higher in hypovitaminosis D children than normal vitamin D children (18.6 ± 8.81 vs 14.8 ± 8.95%; *P*-value = 0.04). Children with hypovitaminosis D had higher mean PTH levels (4.34 ± 1.38 vs. 3.78 ± 1.25 pmol/L; *P*-value = 0.04); however and overall, plasma 25(OH)D and PTH were not found to be significantly correlated (*r* = −0.12; *P*-value = 0.13) (Figure 3). Other factors, such as age, sex, household income, birth weight, medical history, sun exposure time, plasma calcium, phosphate, and magnesium were not different between children with hypovitaminosis D and vitamin D sufficiency (Table 1). In multivariate analysis, high BMI percentile [OR(95%CI) = 1.03 (1.01, 1.06); *P*-value = 0.01] and high PTH concentration [OR(95%CI) = 1.69 (1.06, 2.68); *P*-value = 0.03] were the variables associated with 25(OH)D level < 75 nmol/L (Table 2).

**Table 1:** **Univariate analysis of investigated parameters, according to vitamin D status (n = 159)**

**Parameters**	**Hypovitaminosis D (< 75 nmol/L)**		**Vitamin D sufficiency (≥ 75 nmol/L)**		***P*** **-value**	**Crude OR (95% CI)**
	**n**	**Mean ± SD or number (%)**	**n**	**Mean ± SD or number (%)**		
Age (years)	126	9.86 ± 1.54	33	9.79 ± 1.62	0.82	1.03 (0.81, 1.32)
Female sex	126	87 (69.0%)	33	19 (57.6%)	0.22	1.64 (0.75, 3.61)
Household income	120		31			
≤ 20,000 THB/month		52 (43.3%)		15 (48.4%)	0.61	1
> 20,000 THB/month		68 (56.7%)		16 (51.6%)	-	1.23 (0.56, 2.71)
Income(I):expense(E)	94		25			
I < E		27 (28.7%)		4 (16%)	0.54	1.52 (0.40, 5.78)
I ≅ E		36 (38.3%)		14 (56%)	0.30	0.58 (0.21, 1.62)
I > E		31 (33%)		7 (28%)	-	1
Birth weight	108		28			
< 2.5 kilograms		7 (6.5%)		4 (14.3%)	0.19	0.42 (0.11, 1.54)
≥ 2.5 kilograms		101 (93.5%)		24 (85.7%)	-	1
Allergic diseases	122	20 (16.4%)	32	4 (12.5%)	0.59	1.37 (0.43, 4.34)
Hospitalization	122	40 (32.8%)	31	7 (22.6%)	0.28	1.67 (0.67, 4.21)
Feeding during the first	120		31			
6 months						
Breastfeeding only		39 (32.5%)		7 (22.6%)	-	1
Infant formula only		11 (9.2%)		5 (16.1%)	0.17	0.40 (0.11, 1.49)
Combination		70 (58.3%)		19 (61.3%)	0.39	0.66 (0.26, 1.71)
Sun exposure						
%BSA exposed x	94	238 ± 388	29	179 ± 266	0.45	1.00 (0.999, 1.002)
hrs/wk						
Hours/wk	96	8.1 ± 9.9	29	6.5 ± 8.0	0.43	1.02 (0.97, 1.08)
BMI percentile	126	56.7 ± 33.9	33	42.6 ± 36.0	0.04	1.01 (1.00, 1.02)
Waist circumference (cm)	126	66.3 ± 11.5	33	62.3 ± 9.1	0.07	1.04 (0.997, 1.08)
Waist-to-hip ratio	126		33			
< 0.84		66 (52.4%)		22 (66.7%)	-	1
≥ 0.84		60 (47.6%)		11 (33.3%)	0.15	1.82 (0.81, 4.06)
Body fat percentile	126	36.9 ± 37.8	33	24.7 ± 36.0	0.10	1.01 (0.998, 1.02)
Bone mass (kg)	117	1.45 ± 0.59	31	1.33 ± 0.48	0.29	1.47 (0.72, 3.04)
Muscle mass (kg)	117	28.2 ± 7.27	31	26.2 ± 6.61	0.18	1.04 (0.98, 1.11)
Corrected calcium (mmol/L)	125	2.37 ± 0.06	33	2.36 ± 0.05	0.73	3.06 (0.01, 1943)
Phosphate (mmol/L)	125	1.39 ± 0.16	33	1.40 ± 0.14	0.98	0.96 (0.08, 11.5)
Mg (mmol/L)	125	0.85 ± 0.05	33	0.86 ± 0.05	0.42	0.04 (0.01, 122)
PTH (pmol/L)	126	4.34 ± 1.38	33	3.78 ± 1.25	0.04	1.40 (1.02, 1.94)
Albumin (g/L)	125	45.9 ± 2.36	33	45.9 ± 2.37	0.98	1.00 (0.85, 1.18)

**Table 2:** **Multivariate analysis of factors associated with plasma 25-hydroxyvitamin D < 75 nmol/L (n = 159)**

	**B**	**Adjusted OR (95% CI)**	***P*** **-value**
Birth weight			
< 2.5 kilograms	−0.95	0.39 (0.08, 1. 80)	0.23
≥ 2.5 kilograms	-	1	-
Feeding during the first			
6 months			
Breastfeeding only	-	1	-
Infant formula only	−1.56	0.21 (0.04, 1.17)	0.07
Combination	−1.01	0.37 (0.11, 1.25)	0.11
BMI percentile	0.03	1.03 (1.01, 1.06)	0.01
Body fat percentile	0.00	1.00 (0.97, 1.02)	0.75
Muscle mass (kg)	−0.07	0.93 (0.85, 1.03)	0.15
PTH (pmol/L)	0.52	1.69 (1.06, 2.68)	0.03

## Discussion

This study evaluated the vitamin D status of healthy children in Bangkok, Thailand, which is located in the central region of the country. The findings of this study show that 79.2% of healthy schoolchildren between 6 to 12 years of age have hypovitaminosis D (< 75 nmol/L), with vitamin D insufficiency being observed in about two-thirds (59.7%), and vitamin D deficiency occurring in about one-fifth (19.5%). These results indicate that hypovitaminosis D occurs in people living at low latitude with almost year round sunlight, which is similar to other countries located in abundant sunshine areas [21,22]. Sunlight exposure is a major source of vitamin D, but we were not able to predict vitamin D status from questionnaire questions relating to amount of sun exposure, which likely results from the questionnaire not being validated.

There are studies worldwide that show children with low vitamin D status, which range from 7-72% (cut-off < 50 nmol/L) and 26-98% (cut-off < 75 nmol/L) [20]. This wide range of prevalence can result from variations between different research methods used, between different laboratories using the same method, as well as from different countries located in various latitudes. In tropical countries, school-aged children had prevalence rates of low vitamin D status of 56.6%, 28%, and 68% in Colombia (4°N, 5–12 yrs), Mexico (19°N, 6–12 yrs), and Puerto Rico (18.3°N, < 18 yrs), respectively when using a cut-off value of 25(OH)D < 75 nmol/L [23-25]. However, prevalence rates of 10%, 10%, and 24% were shown when using a cut-off value of 25(OH)D < 50 nmol/L), respectively. In Malaysia (3.09°N), the prevalence of low vitamin D status in children aged 7–12 years was very high (72.3% by using a cut-off value of < 50 nmol/L), as compared to other tropical countries [16]. Recent study of 6–12.9 year old healthy Thai children from four regions (i.e., central, north, northeast, and south) reported a prevalence of vitamin D deficiency of 52.2% in urban (n = 101) and 29.2% in rural areas (n = 217), using a cut-off value of < 50 nmol/L; results which are higher than the results from our study [26]. Another study from Northeast Thailand found that 4% of school-aged children (6.0 to 14.0 yrs, n = 529) had a serum 25(OH)D level below 50 nmol/L, with boys having a statistically significantly higher serum 25(OH)D than girls [27]. Subjects from different parts of the country and the use of different vitamin D assays are possible explanations for the variations in these results. UV irradiance varies in different regions of Thailand, with the southern part recording the highest levels, the northern region the lowest, and the northeast and central regions occupying the middle level [13].

Other studies of various pediatric conditions in the same region of the country as our study have demonstrated that 71.2% of HIV-positive adolescents (12–20 yrs) and 64% of asthmatic children (6–18 yrs) had serum 25(OH)D levels below 75 nmol/L. Moreover, 24.7% of HIV-positive adolescents and 19.2% of asthmatic children had serum 25(OH)D levels below 50 nmol/L, findings which appear to be in a similar range as the findings in our study [28,29]. Another study in HbE/β thalassemia (5.9-14.1 yrs) found a higher prevalence of low vitamin D status when compared with other studies (patients with 25(OH)D levels below 50 nmol/L and 75 nmol/L were 23.9% and 90.0%, respectively) [30].

The finding that children with hypovitaminosis D had a higher BMI percentile than those with a normal vitamin D status is consistent with previous studies that found lower 25(OH)D concentrations in higher BMI children [31-34]. In contrast, a recent study that was performed on younger children did not find the association between BMI and vitamin D status [35]. High BMI associated with vitamin D insufficiency is likely due to a higher body fat content. In our study, although the difference in body fat percentile in the hypovitaminosis D group and the normal vitamin D group was not significant, the body fat percentile tended to be higher in the hypovitaminosis D group. The potential mechanism of low 25(OH)D concentration in high body fat subjects could be: 1) sequestration of vitamin D by adipose tissue; 2) adipose tissue has both vitamin D receptors and 1-α-hydroxylase enzymes, which can synthesize 1,25-dihydroxyvitamin D; and, 3) vitamin D may regulate adipose tissue mass, differentiation, and metabolism; consequently, low vitamin D status might contribute to obesity [36,37]. Another explanation could be that the obese subjects had less exposure to UV radiation [38]; however, we did not find a difference in sun exposure between subjects who were obese or overweight and subjects who were normal or underweight.

In this study, no differences in plasma levels of calcium and phosphate in these two groups of children were shown, but we found significantly higher PTH levels in children with hypovitaminosis D, which was consistent with previous studies [39,40]. Although PTH levels are primarily regulated by ionized calcium and not directly by the level of vitamin D [39], it is well known that low 25(OH)D levels are associated with elevated PTH levels [41]. In addition, the estimation of optimal 25(OH)D concentration has been defined as the concentration that maximally suppresses the parathyroid hormone level [42].

This study has some inherent limitations. This was a single-center study that was undertaken in a limited geographical area – Bangkok, Thailand. The study participant sample reflects an unbalanced gender representation (predominantly females), which may not accurately represent or reflect the gender demographics of children in other areas of Thailand. Without long-term follow-up and additional larger studies it will be difficult to correlate the findings in this study with the onset and/or prevalence of other chronic diseases. We did not elucidate upon how vitamin D is acquired from diet for the following reasons: 1) only a limited amount of information regarding vitamin D content in Thai foods is available [26]; and, 2) a validated food frequency questionnaire and nutrient calculation program for vitamin D in Thai foods are also not currently available.

## Conclusions

The prevalence of hypovitaminosis D in healthy children of Bangkok, Thailand is very high despite their exposure to sunlight. A recommendation for vitamin D supplementation and/or vitamin D fortification of foods should be considered for purposes of maintaining normal vitamin D status in schoolchildren, even in tropical countries like Thailand. Additional randomized clinical trials should be conducted to assess the role of vitamin D in extraskeletal outcomes.
